# Disseminating research findings using a massive online open course for maximising impact and developing recommendations for practice

**DOI:** 10.1186/s12904-020-00564-7

**Published:** 2020-04-22

**Authors:** Nancy Preston, Jeroen Hasselaar, Sean Hughes, Alex Kaley, Lisa Linge-Dahl, Ildiko Radvanyi, Phil Tubman, Karen Van Beek, Sandra Varey, Sheila Payne

**Affiliations:** 1 International Observatory on end of Life Care, Lancaster University, Lancaster, UK; 2 Department of Anaesthesiology, Pain and Palliative Medicine, Radboud University Medical Center, Nijmegen, The Netherlands; 3 Lancaster University, Lancaster, UK; 4 Department of Palliative Care, University Hospital Bonn, Bonn, Germany; 5 University of Pécs Medical School, Pécs, Hungary; 6 Department of Radiation-Oncology and Palliative Medicine, University Hospital Gasthuisberg, Leuven, Belgium

**Keywords:** Integrated, Palliative care, E-learning, Recommendations

## Abstract

**Background:**

Developing recommendations for how we deliver healthcare is often left to leading experts in a field. Findings from the Integrated Palliative Care in cancer and chronic conditions (InSup-C) study, which aimed to identify best practice in integrated palliative care in cancer, chronic obstructive pulmonary disease (COPD) and heart failure, led to recommendations developed through an expert consultation process. We also wanted to develop these recommendations further with participants who were largely clinicians and members of the public.

**Methods:**

Results from the InSup-C study were disseminated through a three-week massive open online course (MOOC) which ran in 2016, 2017 and 2019. The first course helped develop the final recommendations, which were ranked by MOOC participants in the subsequent courses. MOOC participants were predominantly clinicians, but also academics and members of the public. They rated how important each recommendation was on a 9 point scale (9 most important). Descriptive statistics were used to analyse the ratings. The results were compared to findings from the consultation.

**Results:**

Five hundred fifteen completed the last part of the course where the recommendations were ranked, of which 195 (38%) completed the ratings. The top recommendations related to: need to expand palliative care to non-malignant conditions; palliative care needs to include different dimensions of care including physical, psychological and spiritual; policies and regulations assessments should be made regularly; palliative care integration should be mandatory; and there should be greater availability of medicines. These differed compared to the top ranked recommendations by the consultation panel in relation to the importance of leadership and policy making. This may indicate that clinicians are more focused on daily care rather than the (inter) national agenda.

**Conclusions:**

Whilst both sets of recommendations are important, our study shows that we need to include the views of clinicians and the public rather than rely upon leading expert opinion alone. To keep recommendations fresh we need both the input of clinicians, the public and experts. When disseminating findings, MOOCs offer a useful way to gain greater reach with clinicians and the public, and importantly could be a vehicle to validate recommendations made by leading expert panels.

## Background

In 2014 the World Health Assembly passed a resolution urging governments to integrate palliative care into existing health care services [1]. However, it is unclear which are the best models to achieve this [2–4]. A variety of guidelines are available but these have largely been generated through consensus meetings with a limited number of experts, although some have included results of systematic literature reviews and consensus methods [5–7].

The authors were part of a Framework Programme 7 (FP7) European Commission funded study, called InSup-C, which was designed to evaluate best practice in integrating palliative care services in five countries: Netherlands, Belgium, Germany, Hungary and the United Kingdom (UK) [8–10]. Using multiple embedded case study methods, it aimed to find out the best ways to deliver care to people who have advanced cancer, heart failure or lung disease as they come towards the end of their lives. We included 23 integrated palliative care initiatives across the five countries to identify factors associated with good practice in integrating palliative care services.

We presented our findings to a group of international experts and key stakeholders from Europe in 2016. This consensus group then prepared some recommendations based upon consensus methods [11]. However, very few clinicians were involved and those that were, were in senior roles. Hence, we wanted to assess the value of previously generated recommendations by gaining the perspectives of a greater number of people involved in palliative care but especially clinicians.

### Integration of palliative care may occur at three levels

Macro - incorporation of palliative care into national health care strategies and resource allocation plans;

Meso - inclusion of palliative care into regional, local and organisational health care services;

Micro - working at the level of specific patients and families, ensuring that palliative care operates in association with other medical disciplines such as oncology, neurology and geriatrics so that patients experience seamless care [12].

## Methods

This paper seeks to examine the congruence between participant’s opinions enrolled on a MOOC about key recommendations for the further integration of palliative care at three levels: micro, a meso and a macro.

### Design of the study

As part of the dissemination of the InSup-C project we ran a free online course called Palliative Care Making it Work [8]. This Massive Open Online Course (MOOC) ran over 3 weeks and included various activities for the participants including an introduction to Palliative Care, how to integrate it and exemplars of best practice.

#### Effective practice

In the final week of the MOOC, participants were invited to take part in a questionnaire survey which was developed for this study (see Supplementary file), where participants were presented with recommendations from the InSup-C project, and invited to rate each item in terms of its importance. The survey took approximately 15–20 min and could be completed by anyone taking part in the MOOC. Taking part in the independent survey was entirely optional and MOOC participants were not incentivised to do so, either through financial incentives or additional course credits.

The Integrated Palliative Care MOOC was delivered on three separate occasions (October 2016, June 2017 and June 2018). The MOOC 1 (2016) independent survey presented participants with 11 recommendations and was taken directly from the findings of the InSup-C project. MOOCs 2 & 3 (2017/18) were delivered following a consultation workshop attended by international policy makers, including national and international experts in palliative care, in September 2016. The independent survey for MOOCs 2 & 3 therefore presented participants with an additional 15 recommendations, integrating the MOOC 1 survey recommendations and findings from the consultation workshop. This is in keeping with the Delphi method which advocates an iterative process for building consensus and congregating expert opinion [13, 14].

As part of the independent survey, participants were invited to rate the importance of each recommendation item using a Likert scale of 0–9 (where 0 indicated lowest and 9 indicating highest priority). Open comments on the items were possible.

### Sample and settings

The integrated palliative care MOOC was made available to the general public through the Futurelearn platform [15]. Futurelearn is a private company, which offers a diverse selection of courses from leading universities and cultural institutions from around the world. The MOOC was promoted internationally via the Futurelearn platform, and was completed by participants from 148 countries worldwide. No eligibility restrictions were placed on enrolment, and the course was intended for anyone with an interest in palliative care.

A total of 9682 people enrolled on the Integrated Palliative Care MOOC called ‘Palliative Care: Making it work’, which was delivered on three separate occasions (October 2016, June 2017 and June 2018). The recommendations survey was one of the last steps of the 3 week course. As with many MOOCs only about small proportion (10–20%) of those who enrol completed the MOOC [16]. For MOOC 1 (6489 enrolled), 794 people reached the recommendations stage of the MOOC and 460 completed the survey (58%). These results, together with the results from the consensus work shop [11], formed the definitive set of recommendations for MOOC 2 and 3. For MOOCs 2 and 3 (3193 enrolled) a total number of 515 people reached the part of the course containing the recommendations survey. One hundred ninety-five completed the survey (representing a 38% response rate).

In this paper we report on findings from the independent recommendations survey conducted for MOOCs 2 and 3 (2017/18) as these two surveys asked participants to rank an identical set of recommendations and therefore offer comparable data for analysis.

#### Demographic details of participants

When students enrolled on the MOOC, they were invited to complete a short set of questions regarding their sociodemographic background (e.g. gender, age, highest qualification and occupation). This was optional and MOOC participants were not obliged to give this information in order to complete the course. Of the 1293 people who accessed the independent survey, around 22% of those provided responses to the demographic questionnaire. While we cannot, therefore, report on the demographic characteristics for the entire survey sample, by matching the data of those participants who accessed the independent survey with those who completed the demographic questionnaire, we are able to make some general observations about the participants, based on the information given. The majority of participants were women (around 87%) and people in mid to later life appear to represent the dominant age groups (68%). The most commonly reported profession was health and social care (73%) with the majority of participants (83%) educated to degree level or above. Just over half of respondents (54%) were from the United Kingdom, most closely followed by other predominantly English speaking countries, including Australia (8%) Canada (5%) and the US (3%). However, we are unable to analyse the data according to these variables.

### Data collection and analysis

We report descriptive statistics for the survey items. For each item, we report mean agreement to determine the highest ranked items and standard deviation (SD) to determine the degree of consensus. Respondents made very few narrative comments, but these helped to clarify recommendations.

The data from the independent survey reported in this paper were undertaken as part of the MOOC online course and were consultative in nature. As such, we did not seek formal research ethics approval, as this was not required for this activity, and is congruent with other Transparent expert consultation (TEC) studies [17]. Participants were informed that their data would be anonymised and stored according to Lancaster University’s data protection policy and in accordance with general data protection regulations (GDPR), prior to commencement of the survey.

## Results

The recommendations were attributed to three levels.

- macro - national/international level
- meso - organisational/institutional level
- micro - interactions between patients, families and social care professionals

### Ranking of recommendations

Following the survey, analysis of the degree of consensus on all recommendations showing mean and SD are displayed in Tables 1, 2 and 3, across Macro, Meso and Micro levels. Whilst MOOC participants gave high priority to all of the recommendations listed in the independent survey (typically a score of between 7 and 9) our analysis of the degree of consensus did enable us to rank the recommendations in order of importance, relative to one another and the top 5 recommendations are listed in Table 4.

**Table 1:** Recommendations for Integrated Palliative Care at Macro Level

No.	MACRO Recommendations	Mean	SD	Overall Ranking
6	National palliative care regulations and policies should be extended to apply to all patients with palliative care needs, not just those with cancer.	8.59	0.94	1
3	Palliative care regulations and policies should be extended to apply to non-cancer patients as well (for example COPD, heart failure and dementia).	8.52	1.11	3
13	Palliative care should be integrated into mandatory education for undergraduate medical, health and social care professionals.	8.42	1.21	5
14	Continuing professional development for all health and social care professionals should include coverage of integrated palliative care.	8.26	1.07	9
5	For integration to work, new and creative ways of securing resources and specific funding should be established which can support the palliative care infrastructure.	7.95	1.28	18
10	There needs to be national level strategic lobbying to develop and fund better integrated palliative care.	7.95	1.39	19
16	Disease/condition specific national policies should integrate palliative care.	7.95	1.30	20
17	There is a need for strong leadership to advocate for integrated palliative care.	7.94	1.51	21
18	There is a need to invest in the development of future integrated palliative care leadership skills.	7.88	1.44	23
15	Social care should be part of integrated palliative care.	7.51	1.22	26

**Table 2:** Recommendations for Integrated Palliative care at Meso Level

No.	MESO Recommendations	Mean	SD	Overall Ranking
4	The digital transfer of information should be integrated within and across different palliative care services and general services including community and hospital teams, and patients and families.	8.36	1.09	7
2	An information hub, (online or a face-to-face central resource for the coordination of information exchange), with a care co-ordination team should be established to contribute to the integration of palliative care services across the area.	8.12	1.28	12
21	Raise awareness of integrated palliative care for senior managers and policy makers.	8.08	1.26	13
1	Outcome measures to assess quality of integrated palliative care services should be developed.	8.05	1.17	14
23	Outcomes of integrated palliative care should be audited and benchmarked.	8.05	1.25	15
12	Develop alliances within and between health care sectors to build better integration.	8.01	1.21	17

**Table 3:** Recommendations for Integrated Palliative Care at Micro level

No.	MICRO Recommendations	Mean	SD	Overall Ranking
25	Integrated palliative care should encompass different dimensions of care including physical, psychological and spiritual aspects.	8.58	0.95	2
26	Integrated palliative care should involve assessments which are regularly updated and shared with other healthcare professionals within the care team.	8.45	0.97	4
22	Access to readily available and affordable essential medicines are necessary for integrated palliative care.	8.42	1.20	6
9	Develop systems that provide adequate out-of-hours integrated palliative care so that health care practitioners can maintain their work/life balance.	8.30	1.21	8
24	Raise public awareness about palliative care and its integration with healthcare.	8.24	1.13	10
19	Establish needs based referral systems to guide timely referrals to integrated palliative care.	8.16	1.17	11
8	Clinical protocols should be introduced to ensure integration of palliative care services for patients and families regardless of the setting where they are treated.	8.04	1.22	16
20	Establish a single point of contact for integrated palliative care at local level.	7.91	1.58	22
7	Building of informal relationships between health professionals are a foundation for formal structures which are pivotal for the integration of palliative care.	7.81	1.43	24

**Table 4:** Top 5 Recommendations across all levels (MACRO, MESO and MICRO)

No.	Recommendation	Mean	SD
6	National palliative care regulations and policies should be extended to apply to all patients with palliative care needs, not just those with cancer/ Palliative care regulations and policies should be extended to apply to non-cancer patients as well (for example COPD, heart failure and dementia).	8.59/ 8.52	0.94/ 1.11
25	Integrated palliative care should encompass different dimensions of care including physical, psychological and spiritual aspects.	8.58	0.95
26	Integrated palliative care should involve assessments which are regularly updated and shared with other healthcare professionals within the care team.	8.45	0.97
13	Palliative care should be integrated into mandatory education for undergraduate medical, health and social care professionals.	8.42	1.21
22	Access to readily available and affordable essential medicines are necessary for integrated palliative care.	8.42	1.2

Overall, all recommendations indicated high or medium level consensus on their importance [11]. Two recommendations (RECs) (RECs 3 and 6 – macro level) were very similar and ranked highest so are treated as one which related to palliative care services needing to be broadened to beyond cancer. The next highest recommendation was about ensuring palliative care encompassed all dimensions of care including physical, spiritual and psychological (REC 25 - micro level). The third highest related to the need for regular assessment (REC 26 – micro level). The fourth was the need for palliative care to be mandated into undergraduate education of health care professionals (REC 13 – macro level). The fifth highest was about making essential medicines affordable and available (REC 22 – micro level). There was a greater diversity in responses to a number of macro level statements no. 5, 10, 16, 17, 18 and two Micro level statements no.7 and 20, as indicated by larger SDs. This suggests that there was less agreement regarding the importance of these recommendations.

#### Policies need to extend palliative care beyond cancer (RECs 3 and 6)

Members of the MOOC came from 148 countries and, for some, the idea of extending palliative care beyond cancer was novel. This may be why introducing the idea of extending palliative care beyond cancer was rated so highly. Even for those who already recognised this need, they may have felt in day-to-day practice services were still predominantly aimed at patients with cancer hence they felt the need to reinforce this recommendation.

#### Including different dimensions of care including physical, spiritual and psychological

Physical care has become increasingly predominant in palliative care and perhaps the MOOC raised the profile of non-physical care with participants recognising that this also needed prioritising. This was endorsed by using the WHO definition of palliative care in the MOOC which may have been new to some of the participants who included generalists as well as specialists in palliative care.

#### Need for regular assessments shared with the care team

For the clinicians in the MOOC, regular assessment may have been prioritised to maintain good daily care on a micro-level. The need for regular assessment was not highlighted in the MOOC but gaining greater involvement with the multidisciplinary team was. Sharing results of assessments may have been linked to the importance of working in multi-disciplinary contexts.

#### Education

Education was considered a key priority by MOOC participants. For them, palliative care should be integrated into mandatory education for undergraduate medical, health and social care professionals (REC 13) (ranked 5th in order of importance). Also regarded as important (although lower ranked) was the inclusion of integrated palliative care in the continuing professional development of health and social care professionals (REC 14). This suggests that MOOC participants regarded education about integrated palliative care to be a higher priority for those at the beginning of their health and social care careers.

#### Access to and affordability of essential medications

Education about access to opioids world-wide was covered extensively in the MOOC and may have been novel, particularly for the UK participants who made up about 50% of the cohort. Equally they may have been reflecting on the timely provision of medicines when they may be required at short notice as a patient’s condition deteriorates.

## Discussion

The ranking of the recommendations about how to integrate palliative care are broadly similar to those produced through the consensus meeting [11]. However the MOOC participants who were largely clinicians prioritised the need for education. This may be because they recognised their own needs whereas the members of the consensus group were already highly informed about the need for palliative care and were focussing on how to develop palliative care at a national or international level [18, 19]. If clinicians recognise that they need more education about how to integrate palliative care, then this need should be rated higher with policy makers, such as those at the consensus meeting, as without policy makers’ support change will not happen. The members of consensus group highlighted a greater need to raise public awareness [20], which was a lesser concern for the MOOC participants indicating that they were perhaps more focussed on the need to change day to day care, rather greater changes across society. Another area of difference was in relation to leadership. Participants from the consensus group, who could be argued are already in leadership roles, rated the need higher (REC 17, 18) than MOOC participants. Palliative care has evolved as a largely social movement with greater focus on developing relationships as a means to developing palliative care [10]. The emphasis has been on charismatic champions, rather than other types of leadership, so those conducting day to day care will be operating more on this relational level than seeing leadership as a necessary priority.

MOOC participants gave less priority in general to policy recommendations. One way to interpret this is may be that how participants view issues relating to policy is influenced by the level at which they operate. For example, if you operate at a more strategic or national level then policy making would be perceived as having a greater significance than it would for service or ‘ground level’ practitioners. This was similar to recommendations about audit and outcome measures where the consensus group gave this higher priority. Evidence and audit are needed to influence policy and allocation of resources [21] whereas relationships are potentially more important to influence change in daily practice [10]. Even though MOOC participants recognised the need to establish these informal relationships, (REC 6) they placed greater emphasis on developing alliances within and between health care sectors (REC 11). This was similar to the consensus group, with both groups recognising the need for multiple agency involvement to deliver effective integrated palliative care.

There was a higher priority in relation to the need for integrated palliative care to encompass the different dimensions of palliative care namely physical, psychological and spiritual aspects [1]. This was echoed in some of the feedback from MOOC participants during the course, which demonstrated these were new ideas for some, as many were not specialist palliative care practitioners. This reminds us that palliative care occurs outside of specialist care contexts and more effective involvement and work with generalists providing palliative care is required if change is to be achieved. The MOOC, which included people from 148 countries and from a large range of disciplines, was therefore a useful mechanism to engage people in developing recommendations.

There is a great need for access to good out of hours palliative care [22] services, but this was recognised more by the MOOC participants than by the members of the consensus group. Once again, this may reflect the daily experience of staff. From our own research on integrated palliative care [9, 10], we found that in some countries, this was only achieved by practitioners giving personal contact numbers and being permanently ‘on call’. Whilst this was not true in all countries, it may go some way to explaining why the MOOC participants rated maintaining a work life balance (REC 9) higher than those at the consensus workshop.

The contrast between the day-to-day needs of practitioners compared to the ratings from the consensus group, was evident in relation to accessibility and affordability of medicines (REC 22), which was rated higher by MOOC participants. This may also be reflective of the experiences of MOOC participants, drawn from around the world and the wide variability in access to medicines. The accessibility and affordability of medicines was included as teaching topic on the MOOC. This may have been one of the first times MOOC participants, particularly those from the UK with a readily available supply of opiates, had realised access was not universal. This contrasts to the consensus group who rated changes at national level through lobbying to develop and gain better funding for integrated palliative care (REC 10) or develop new innovative ways to secure funding (REC 5) as higher. This suggests that MOOC participants concerns around funding are micro-level rather than macro-level related, in contrast to consensus group of international experts.

### Study strengths and limitations

This is the first independent survey that specifically elicits practitioners’ (and other professionals’) views on integrated palliative care, and compares this large sample with the views of experts [9]. Our study is innovative in that it incorporated a wide range of views on recommendations and priorities for integrated palliative care rather than just the views of experts. Further, all participants were well informed having engaged in a course about integrated palliative care. However, they may not have been representative of all palliative care workers as they had self-selected to attend the course. Even though all participants (MOOC and experts) had similar information about the outcomes of the primary study, their own experiences appeared to influence how they ranked the recommendations. A limitation of the study was that we did not have full demographic information for each participant, although we were able to make some general observations about the characteristics of the participants, based on the socio-demographic information given at the point of enrolment. However, we weren’t able to analyse specific variables such as country of origin, role, or gender. Also, as the recommendations came at the end of the course it is unclear if participants ranked items in a way to ‘please’ the course tutors by ranking certain topics higher which they perceived were presented as important on the course [23]. However this potential limitation was mitigated because the ranking was anonymous.

## Conclusions

Our findings suggest that although recommendations were similar for MOOC participants and those generated at the consensus workshop, there were some important differences. The MOOC gave a platform to gain a broader range of opinions, including those of clinicians, which is useful when making recommendations for advancing palliative care policy and practice. The MOOC is a useful mechanism for gaining greater dissemination of research but also as a vehicle for producing recommendations.

## Supplementary information

**Additional file 1.** Questionnaire developed for this study

## Data Availability

All data are archived at Lancaster University and may be obtained from the first author.

## Abbreviations

InSuP-C: Integrated Palliative Care in cancer and chronic conditions
COPD: Chronic obstructive pulmonary disease
MOOC: Massive open online course
FP7: Framework Programme 7
UK: United Kingdom
SD: Standard deviation
TEC: Transparent expert consultation
GDPR: General data protection regulations
RECs: Recommendations
