# Supplementary material for: Disseminating research findings using a massive online open course for maximising impact and developing recommendations for practice
Source: BMC Palliat Care. 2020 Apr 22;19:54. doi: 10.1186/s12904-020-00564-7 (PMC7178937; doi:10.1186/s12904-020-00564-7)
Supplement: Supplementary file 1 — Additional file 1. Questionnaire developed for this study [file 12904_2020_564_MOESM1_ESM.docx]

Please read each of the following statements. Drawing on your own experience, whether personal or professional, please indicate how important you consider each one of the following statements about integrated palliative care.

Please rank with 1-9, where 1 is not important and 9 is extremely important.

|  | 1 | 2 | 3 | 4 | 5 | 6 | 7 | 8 | 9 |
| --- | --- | --- | --- | --- | --- | --- | --- | --- | --- |
| 1. Outcome measures to assess quality of integrated palliative care services should be developed. | ⃝ | ⃝ | ⃝ | ⃝ | ⃝ | ⃝ | ⃝ | ⃝ | ⃝ |
| 1. An information hub, (online or a face-to-face central resource for the coordination of information exchange), with a care co-ordination team should be established to contribute to the integration of palliative care services across the area | ⃝ | ⃝ | ⃝ | ⃝ | ⃝ | ⃝ | ⃝ | ⃝ | ⃝ |
| 1. Palliative care regulations and policies should be extended to apply to non-cancer patients as well (for example COPD, heart failure and dementia) | ⃝ | ⃝ | ⃝ | ⃝ | ⃝ | ⃝ | ⃝ | ⃝ | ⃝ |
| 1. The digital transfer of information should be integrated within and across different palliative care services and general services including community and hospital teams, and patients and families. | ⃝ | ⃝ | ⃝ | ⃝ | ⃝ | ⃝ | ⃝ | ⃝ | ⃝ |
| 1. For integration to work, new and creative ways of securing resources and specific funding should be established which can support the palliative care infrastructure. | ⃝ | ⃝ | ⃝ | ⃝ | ⃝ | ⃝ | ⃝ | ⃝ | ⃝ |
| 1. National palliative care regulations and policies should be extended to apply to all patients with palliative care needs, not just those with cancer. | ⃝ | ⃝ | ⃝ | ⃝ | ⃝ | ⃝ | ⃝ | ⃝ | ⃝ |
| 1. Building of informal relationships between health professionals are a foundation for formal structures which are pivotal for the integration of palliative care. | ⃝ | ⃝ | ⃝ | ⃝ | ⃝ | ⃝ | ⃝ | ⃝ | ⃝ |
| 1. Clinical protocols should be introduced to ensure integration of palliative care services for patients and families regardless of the setting where they are treated. | ⃝ | ⃝ | ⃝ | ⃝ | ⃝ | ⃝ | ⃝ | ⃝ | ⃝ |
| 1. Develop systems that provide adequate out-of-hours integrated palliative care so that health care practitioners can maintain their work/life balance. | ⃝ | ⃝ | ⃝ | ⃝ | ⃝ | ⃝ | ⃝ | ⃝ | ⃝ |
| 1. There needs to be national level strategic lobbying to develop and fund better integrated palliative care. | ⃝ | ⃝ | ⃝ | ⃝ | ⃝ | ⃝ | ⃝ | ⃝ | ⃝ |
| 1. Clarification of the language and terms used to describe integrated palliative care and associated services is needed. | ⃝ | ⃝ | ⃝ | ⃝ | ⃝ | ⃝ | ⃝ | ⃝ | ⃝ |
| 1. Develop alliances within and between health care sectors to build better integration. | ⃝ | ⃝ | ⃝ | ⃝ | ⃝ | ⃝ | ⃝ | ⃝ | ⃝ |
| 1. Palliative care should be integrated into mandatory education for undergraduate medical, health and social care professionals. | ⃝ | ⃝ | ⃝ | ⃝ | ⃝ | ⃝ | ⃝ | ⃝ | ⃝ |
| 1. Continuing professional development for all health and social care professionals should include coverage of integrated palliative care. | ⃝ | ⃝ | ⃝ | ⃝ | ⃝ | ⃝ | ⃝ | ⃝ | ⃝ |
| 1. Social care should be part of integrated palliative care. | ⃝ | ⃝ | ⃝ | ⃝ | ⃝ | ⃝ | ⃝ | ⃝ | ⃝ |
| 1. Disease/condition specific national policies should integrate palliative care | ⃝ | ⃝ | ⃝ | ⃝ | ⃝ | ⃝ | ⃝ | ⃝ | ⃝ |
| 1. There is a need for strong leadership to advocate for integrated palliative care. | ⃝ | ⃝ | ⃝ | ⃝ | ⃝ | ⃝ | ⃝ | ⃝ | ⃝ |
| 1. There is a need to invest in the development of future integrated palliative care leadership skills. | ⃝ | ⃝ | ⃝ | ⃝ | ⃝ | ⃝ | ⃝ | ⃝ | ⃝ |
| 1. Establish needs based referral systems to guide timely referrals to integrated palliative care. | ⃝ | ⃝ | ⃝ | ⃝ | ⃝ | ⃝ | ⃝ | ⃝ | ⃝ |
| 1. Establish a single point of contact for integrated palliative care at local level. | ⃝ | ⃝ | ⃝ | ⃝ | ⃝ | ⃝ | ⃝ | ⃝ | ⃝ |
| 1. Raise awareness of integrated palliative care for senior managers and policy makers. | ⃝ | ⃝ | ⃝ | ⃝ | ⃝ | ⃝ | ⃝ | ⃝ | ⃝ |
| 1. Access to readily available and affordable essential medicines are necessary for integrated palliative care | ⃝ | ⃝ | ⃝ | ⃝ | ⃝ | ⃝ | ⃝ | ⃝ | ⃝ |
| 1. Outcomes of integrated palliative care should be audited and benchmarked | ⃝ | ⃝ | ⃝ | ⃝ | ⃝ | ⃝ | ⃝ | ⃝ | ⃝ |
| 1. Raise public awareness about palliative care and its integration with healthcare | ⃝ | ⃝ | ⃝ | ⃝ | ⃝ | ⃝ | ⃝ | ⃝ | ⃝ |
| 1. Integrated palliative care should encompass different dimensions of care including physical, psychological and spiritual aspects | ⃝ | ⃝ | ⃝ | ⃝ | ⃝ | ⃝ | ⃝ | ⃝ | ⃝ |
| 1. Integrated palliative care should involve assessments which are regularly updated and shared with other healthcare professionals within the care team | ⃝ | ⃝ | ⃝ | ⃝ | ⃝ | ⃝ | ⃝ | ⃝ | ⃝ |
